# The relationship between talent management and the efficiency of head nurses and senior and middle managers from the educational and medical centers in Tabriz, Iran – a case study

**DOI:** 10.25122/jml-2017-0066

**Published:** 2022-08

**Authors:** Sajad Hadi Chelan, Khalil Alimohammadzadeh, Ali Maher

**Affiliations:** 1 School of Management and Social Sciences, North Tehran Branch, Islamic Azad University, Tehran, Iran; 2 Department of Healthcare Management, School of Management and Social Sciences, North Tehran Branch, Islamic Azad University, Tehran, Iran; 3 Health Economics Policy Research Center, Tehran Medical Sciences Islamic Azad University, Tehran, Iran

**Keywords:** talent management, efficiency, managers, head nurses

## Abstract

Nowadays, organizations understand that they need the best talent to succeed in the complex world economy and survive in a competitive business environment. Therefore, talent management can ensure that each employee with a unique talent or ability will be placed in the correct position. This article aimed to study the relationship between talent management, senior and middle managers, and head nurses from educational health and research centers in Tabriz, in 2016. The target population included senior and middle managers and head nurses from Tabriz University of Medical Sciences, approximately 197 people. The sample for this study was selected based on Morgan's table, which rounds up to 123 people. The Kolmogorov-Smirnov test was used to analyze data, and if data were normal, correlation and regression analysis were performed. There was a significant relationship between talent management and the efficiency of senior and middle management and head nurses from the educational and medical centers in Tabriz. Therefore, when talent management increases, the efficiency level also rises to a noticeable degree. Also, the linear regression showed a linear relationship between talent management as an independent variable and efficiency as a dependent variable. Applying talent management strategies in the management selection process in organizations with demanding environments such as hospitals seems inevitable so that managers with the highest efficiency are hired.

## INTRODUCTION

Nowadays, organizations realize they need the best talents to succeed in the world-complex economy and survive in the competitive business environment. In the meantime, realizing the need to employ, promote and keep talents, organizations found that talents are critical sources which require management to achieve optimal results [1]. Promoting and developing human resources (HR) in organizations, considered the most important source of production, is the secret to an enterprise's survival [2]. The most significant challenge in business is not only technology alone but also the exploitation and election of smart HR together with capable human assets, which is also said to be the main secret for facing challenges and losses in business [3]. Therefore, most scholars and scientists now refer to the concept of talent management. Talent management assures organizations have qualified personnel with appropriate skills to be placed in the right position [4]. One could define talent management as a systematic business process that fills the gap between the current talents of the organization (current situation) and the ones required (optimal situation) in facing the current and future challenges of the organization [5]. Talent management provides tools for managers to appreciate their expectations in dealing with their staff, leading to good employer-employee relationships [6]. Accordingly, the organization gains higher returns out of management investments.

There is little room for doubt that working in hospitals and medical centers has many health issues for employees and individuals. Therefore, research was conducted in this field, namely on the effect of Covid-19 [7–10]. Research indicates that one of the main reasons for stress in the working environment is workload which sometimes leads to depression, and it has a direct relationship with many diseases like cancer [11–18], brain tumors [19], Alzheimer's [20, 21], Parkinson's [22], cyst [23], bone mimicking [24], varices and bleeding risk [25]. To tackle this issue, some say to take a break from working, even for a short time; in many cases, employees have 15–30 minutes to rest or sleep. Many researchers indicate that sleeping could reduce stress [26, 27]. However, there are many other aspects too. Health care strategies are highly significant in many aspects and could enhance qualifications [28]. Nursing studies in the health sector show managers' failure to enhance the participation and performance of staff [29]. The lack of attention of managers to HR capabilities may lead to failure in creating positive organizational changes. The talent management topic has become a necessary and inseparable strategy for most companies and organizations. Many scholars published their work concerning talent management. Scullion & Collings (2011) studied top people attraction criteria in Convergys Co. using interviews and questionnaires [4]. Based on their results, five criteria were specified. Out of these criteria, they discussed about knowledge and education literacy (42%), immediate performance (21%), personal characteristics (especially communication skills) (17%), career background (12%), talent progress potential (8%) of the identification process & keeping talented people in the company to themselves [30]. They concluded that the talent management method in large multinational companies is interwoven in a particular way with the systems, strategies, structure, and organizational goals.

On the other hand, small companies have no specific strategy to identify talented staff. However, they focus instead on activities that keep their immediate personnel, such as competitive rewards, creating progress opportunities, making a friendly environment by delegating suitable posts with individual talents and capabilities, and the appropriate organizational culture of the staff.

Walter *et al*. 2010 studied Philips & Roper's talent management model and career satisfaction concerning the staff of the organization in 28 Indian companies with a staff of 4811 experts [31]. The outcome revealed a relation between the components of this model (such as attraction, appointment, involvement, development and repair, maintenance), career satisfaction, and organizational satisfaction. Among these components, involvement (37%) was the highest, and attraction (9%) represented the lowest share regarding staff satisfaction.

The researchers affirm that one of the main reasons for this organization's success was leaders' particular attention to the development and education of senior managers in the organizations and believe that education in these organizations is an inseparable component of the talent management process. Work enrichment programs in these organizations have been implemented by emphasizing HR in designing and planning methods and training future leaders (training successors). In addition, the talent management process within companies includes different groups, including executives, all managers of different levels, and HR units. Regarding the importance of talent management in managers, talent management has turned into a hot topic in recent years. Investigating TM (talent management) in the nursing field gained importance due to the direct contact between the talent and superior performance of the organization. Many studies showed that emotional treatment could enhance performance even in patients or employees [32–34]. Furthermore, studies indicate that when an organization like a hospital invests in its talents, its revenue greatly increases. Therefore, talents can impact business performance, and nurses who consist of a large number of employees in the hospital could accordingly affect the hospital's performance. Paying attention to talent management in a hospital environment is necessary [35, 36]. In the case of hospitals and training centers, looking at the organization's future mission and perspectives and their need for efficient managers drove us to answer the question of whether there is a relationship between talent management and the efficiency of senior and middle managers as well as nurses from educational, medical and research centers in the city of Tabriz.

As an organization, hospitals are handled by many executive and middle managers, and because these managers play an influential role in increasing efficiency, this could be a suitable environment for our research. Since mood and aging in individuals play a primary role in this field, Vesel *et al*. (2020) conducted research, and their findings indicate that working conditions can affect mental health [37].

Research indicates that efficiency increases when talented employees work in medical places. In hospitals, many procedures or conditions can affect the quality of health of patients, such as magnetic resonance imaging (MRI) [38], positron emission tomography (PET) scan [39–41], radiology [42], pharmaceutics [43–45], pregnancy [46] etc. This study is important because it can add valuable information for senior managers, middle managers, and nurses in training and subsequently impact the efficiency of medical and research centers. In broader aspects, it can lead to the permanent development of the Tabriz University of Medical Sciences and Health Services.

## Materials and Methods

This descriptive study used a survey to depict the current situation, including 198 senior and middle managers and head nurses from educational, medical, and research centers in Tabriz. Based on Morgan's table in this study, 123 people were selected from the above sample. The study was implemented in Tabriz's educational, medical, and research centers and was conducted during the spring/summer of 2016.

### Study Hypotheses

There is a significant relationship between talent management and the efficiency of senior, middle managers, and head nurses from the educational, medical & research centers in Tabriz.

### Technical Hypothesis

There is a significant connection between talent attraction, talent selection, talent enrichment, keeping talent and senior, middle managers, and head nurses in educational, medical & research centers in Tabriz.

### Talent management process model

Several models were presented by different experts and theoreticians in talent management literature. Table 1 shows the talent management process applied in this study. To determine the validity of the questionnaire in this study, some guide masters, consultants, and experts' opinions from the Medical, Education, and Statistics Departments of the University of Tabriz were applied. To determine the reliability of the tools, Cronbach's alpha was used (Table 2). At first, 20 questionnaires were distributed among the subjects. Cronbach's alpha coefficient equaled 0.87. Since the eventuated coefficient exceeded 0.7, the reliability was confirmed. Analyzing data was implemented in two descriptive & inferential data sections, using Likert measuring scales. In the descriptive section, the frequency distribution and indicators of central tendency and dispersion were used. In this study, the Kolmogorov-Smirnov test was used to specify data normality and abnormality. In the case of normal data, the correlation coefficient and regression test were used. The Statistical Package for the Social Sciences (SPSS) software was used to analyze the data.

**Table 1:** The talent management process applied.

**Talent management**	**Talent attraction**	Pay-based competition	Individual performance
		Work-life balance	
		Challenging job	
		The good reputation of an organization for having an effective employer	
	**Talent selection**	Psychological test	
		Behavior interview	
		Personality assessment	
		Work-knowledge test	
	**Talent employment**	Harmony between the job and employed	
		Harmony between the employed and job	
	**Talent development**	Training talents	
		Talents' special career path	
		HR strategic planning	
		Successors training	
	**Keeping talent**	Special compensation service system	
		The transitional style of leadership	

**Table 2:** Cronbach's alpha coefficient for talent management and efficiency.

Concept under study	Dimensions	No. of questions	Cronbach's Alpha	Concept under study
**Connection between talent management & efficiency**	Talent management	27	0.932	0.940
	Efficiency	23	0.813	

## Results

### Descriptive Statistics

The marital status of the participants is mentioned in Table 3. Based on the results, 16.26% were single, and most of the participants (83.74%) were married.

**Table 3:** Distribution of marital status.

Married situation	No. (frequency)	Percent
**Single**	20	16.26
**Married**	103	83.74
**Total**	123	100

Regarding education, based on the outcomes, 3.25% had a diploma, 9.76% had an associate degree, 71.54% had a bachelor and 15.45% a master's degree and higher.

Most participants in the study had a bachelor's degree (71.54%). Furthermore, 24.39% had a work experience of 1–2 years, 38.21% of 3–5 years, 16.26% of 6–10 years, and 21.14% had more than 10 years (Table 4). In Table 5, the distribution was organized based on their work experience in the management position. The results show that 4.05% of people had 1–2 years of managerial experience, 10.60% had 3–5 years, 21.95% had 6–10 years, and 48.77% had 11–20 years while 14.63% had 21–30 years of experience. The maximum frequency was related to people with 11–20 years of management experience, and the least frequency corresponded to people with 1–2 years of experience. In Table 6, the talent management scores of people were assessed. Based on the outcomes, the average score was 93.45, with a standard deviation of 14.02. The lowest score was 81, and the maximum score was 118. Also, the statistical description of the efficiency scores was performed (Table 7). Based on the outcomes, the average score was 44.1 with a standard deviation of 7.59, with the lowest score being 26 and the maximum score 60.

**Table 4:** Educational level.

Education level	Percentage (%)
**Diploma**	3.25
**Associate degree**	9.76
**Bachelors' degree**	71.54
**Master's degree and higher**	15.45

**Table 5:** Management experience.

Statistical indicators of management experience	Number (frequency)
**1–2 years**	5
**3–5 years**	13
**6–10 years**	27
**11–20 years**	60
**20–30 years**	18
**Total**	123

**Table 6:** Descriptive statistics of talent management scores.

Statistical indicators	No.	Average	Standard Deviation	Least	Most
**Talent management**	123	93.45	14.011	82	118

**Table 7:** Efficiency scores.

Statistical indicators	No.	Average	Standard Deviation	Least	Most
**Talent management**	123	44.1	7.59	26	60

### Linear regression between talent management and efficiency indicators

We investigated whether there was a positive relationship between talent management and efficiency indicators.

- Hypothesis H_0_ – no linear relationship between talent management and efficiency;
- Hypothesis H_1_ – a linear relationship between talent management and efficiency.

For this reason, the linear regression test was used using the “enter” method, and the results are illustrated below. Table 8 reveals that the p-value was less than 5%, indicating a linear relationship between talent management (as an independent variable) and efficiency (as a dependent variable). Table 9 shows the preliminary amount and talent management coefficient to predict the rate of change in efficiency. The fixed coefficient value in the model was 2.16, and the performance indices coefficient rate was 0.39. Based on the statistics, both coefficients showed a significant value of less than 5%; therefore, both models remained.

**Table 8:** ANOVA results after studying the linear relation between the two variables.

Model	Sum of Squares	Df	Mean Square	F	Sig.
**Regression 1**	14.700	1	14.700	50.238	0.001
**Residual**	30.291	112	.280	-	-
Total	44.991	113	-	-	-

**Table 9:** Regression test results for determining the coefficient rates of the model between talent management and efficiency.

Model	Unstandardized coefficients		Unstandardized coefficients	T	Sig.
	B	Std.	Beta		
**Constant**	2.160	.208	.553	10.477	0.001
**Total performance indicators**	.390	.064		7.017	0.001

### Main Hypotheses

There is a significant relationship between talent management and the efficiency of senior, middle managers, and head nurses from the educational and medical centers of Tabriz's hospitals.

### The Assumed Statistical hypothesis

Null hypothesis H_0_: ρ≤0 There is no significant relationship between talent management and efficiency of senior, middle managers, and head nurses from educational and medical centers in Tabriz.

Alternative hypothesis H_1_: ρ>0 There is a significant relationship between talent management and efficiency of senior, middle managers, and head nurses from educational and medical centers in Tabriz. Using the relative test, at a confidence level of 99%, the p-value equaled 0.001 and less than 0.01, and consequently, H_0_ at the level beyond 1 percent of H_1_ was confirmed. In other words, at the error level of 1% between talent management and efficiency of senior and middle managers and head nurses in the educational and medical centers in Tabriz with the correlation coefficient of 0.330, the significant positive relation was confirmed and verified.

### Correlation coefficient significance test for the fifth hypotheses

The results after testing the significance of the correlation coefficient for the first (talent attraction), second (talent selection), third (talent employment), fourth (talent development), and fifth (keeping talent) hypotheses will be presented below in Tables 10–15, respectively.

**Table 10:** Results of the main hypothesis correlation coefficient test.

Type of relation	Significance level	R2 Determination coefficient	R Correlation coefficient	Error level	No.	Connection type
**Positive**	0.001	0.0001	0.330	0.05	123	linear

**Table 11:** Test results of the correlation coefficient test of the first hypothesis.

Type of relation	Significance level	R2 Determination coefficient	R Correlation coefficient	Error level	No.	Connection type
**Positive**	0.001	0.16	0.403	0.05	123	linear

**Table 12:** Test results of the correlation coefficient test of the second hypothesis.

Type of relation	Significance level	R2 Determination coefficient	R Correlation coefficient	Error level	No.	Connection type
**Positive**	0.001	0.16	0.335	0.05	123	linear

**Table 13:** Test results of the correlation coefficient test of the third hypothesis.

Type of relation	Significance level	R2 Determination coefficient	R Correlation coefficient	Error level	No.	Connection type
**Positive**	0.001	0.31	0.545	0.05	123	linear

**Table 14:** Test results of the correlation coefficient test of the fourth hypothesis.

Type of relation	Significance level	R2 Determination coefficient	R Correlation coefficient	Error level	No.	Connection type
Positive	0.001	0.006	0.390	0.05	123	linear

**Table 15:** Test results of the correlation coefficient test of the fifth hypothesis.

Type of relation	Significance level	R2 Determination coefficient	R Correlation coefficient	Error level	No.	Connection type
**Positive**	0.001	0.18	0.401	0.05	123	linear

### The assumed statistical hypothesis

Null hypothesis H_0_: ρ≤0 There is no significant relationship between talent attraction, talent selection, talent employment, talent development, keeping talent and efficiency of senior, middle managers, and head nurses from educational and medical centers in Tabriz.

Alternative hypothesis H_1_: ρ>0 There is a significant relationship between talent attraction, talent selection, talent employment, talent development, keeping talent and efficiency of senior, middle managers, and head nurses from educational and medical centers in Tabriz.

By implementing the relative test, at a confidence level of 99%, the p-value equaled 0.001 and less than 0.01, and consequently, it was verified and confirmed through a level beyond 1 percent H_1_. In other words, at the error level of 1% between talent attraction, selection, employment, development and keeping talents and the efficiency of the senior and middle managers and head nurses from the educational and medical hospitals in Tabriz with the correlation coefficient of 0.403, 0.335, 0.545, 0.390 and 0.401, respectively. These are positive and significant relationships that have been confirmed.

## Discussion

The talent management process and successor development should focus on key positions because complex institutions such as hospitals and medical centers are key organizations where hiring qualified staff would be time-consuming and difficult. Key roles in such complex organizations substantially impact their success and should not be left vacant for a long time or in the hands of unqualified people. Otherwise, such hospitals will not be able to compete with other hospitals. Owing to this fact, the key and sensitive positions of the organization are to be left in the hands of talent management programs by using keen techniques. Talent management could play an influential role in keeping talented HR. In the time of “knowledgeable workers”, organizations compete with each other based on their staff's skills and talents.

By attracting and keeping the most talented staff, one has the best tool to ensure the organization's competitive advantage, and in this line, only those organizations with the most talented and hardworking staff are considered among the pioneer ones. Concerning the findings of the current study, one could conclude that applying the talent management process in selecting managers for different levels in hospitals shall lead to a treasure of managers with high performance and potential. When necessary, a qualified manager shall be available. Since executive and managing posts are limited when it comes to hospitals, there shall be a competitive atmosphere among managers that will promote their qualifications in the system to establish their position.

## Conclusion

This paper studied the relationship between talent management and the efficiency of senior managers, middle managers, and head nurses from the educational and medical centers in Tabriz, Iran. It is necessary to apply some strategic talent management techniques in securing a management post in organizations with high complexity, such as hospitals, so only qualified managers can be hired. As the results show, there is a significant relationship between efficiency and talent management. Thus, when talent management increases, so does efficiency as well. According to our objectives, results, and observations during the study, some suggestions and recommendations to increase productivity, career satisfaction, and efficiency of the staff are stated below.Hospitals, senior managers, middle managers, and head nurses should be serious about providing and implementing talent management to create and develop an organizational culture.

Hospitals should pay special attention to talent management and the exact schedule to implement it through strategic methods, objectives, and operational planning. Senior managers, middle managers, and head nurses should try to reflect talent management techniques in their behavior and provide spiritual support through positive methods.

## Personal thanks

The authors are grateful to all the medical practitioners for actively participating in the study.
